# Prognostic role of pre-diagnostic circulating inflammatory biomarkers in breast cancer survival: evidence from the EPIC cohort study

**DOI:** 10.1038/s41416-024-02858-6

**Published:** 2024-09-28

**Authors:** Carlota Castro-Espin, Manon Cairat, Anne-Sophie Navionis, Christina C. Dahm, Christian S. Antoniussen, Anne Tjønneland, Lene Mellemkjær, Francesca Romana Mancini, Mariem Hajji-Louati, Gianluca Severi, Charlotte Le Cornet, Rudolf Kaaks, Matthias B. Schulze, Giovanna Masala, Claudia Agnoli, Carlotta Sacerdote, Marta Crous-Bou, Maria-Jose Sánchez, Pilar Amiano, María-Dolores Chirlaque, Marcela Guevara, Karl Smith-Byrne, Alicia K. Heath, Sofia Christakoudi, Marc J. Gunter, Sabina Rinaldi, Antonio Agudo, Laure Dossus

**Affiliations:** 1 Unit of Nutrition and Cancer, Catalan Institute of Oncology-ICO, L’Hospitalet de Llobregat, Barcelona, Spain; 2 Nutrition and Cancer Group, Epidemiology, Public Health, Cancer Prevention and Palliative Care Program, Bellvitge Biomedical Research Institute-IDIBELL, L’Hospitalet de Llobregat, Barcelona, Spain; 3 Université Paris-Saclay, UVSQ, Inserm, Gustave Roussy, CESP, 94805 Villejuif, France; 4 Nutrition and Metabolism Branch, International Agency for Research on Cancer, World Health Organization, Lyon, France; 5 Department of Public Health, Aarhus University, Bartholins Alle 2, DK-8000 Aarhus C, Denmark; 6 Danish Cancer Institute, Copenhagen, Denmark; 7 Department of Public Health, University of Copenhagen, Copenhagen, Denmark; 8 Department of Cancer Epidemiology, German Cancer Research Center (DKFZ), Heidelberg, Germany; 9 Department of Molecular Epidemiology, German Institute of Human Nutrition, Nuthetal, Germany; 10 Institute of Nutritional Science, University of Potsdam, Nuthetal, Germany; 11 Clinical Epidemiology Unit, Institute for Cancer Research, Prevention and Clinical Network (ISPRO), Villa delle Rose Via Cosimo il Vecchio, 2- 50139 Florence, Italy; 12 Epidemiology and Prevention Unit, Fondazione IRCCS Istituto Nazionale dei Tumori, Via Venezian, 1 - 20133 Milan, Italy; 13 Department of Health Sciences, University of Eastern Piedmont, Via Solaroli 17, 28100 Novara, Italy; 14 Unit of Epidemiology, Local Health Unit of Novara, viale Roma, 7, 128100 Novara, Italy; 15 Department of Epidemiology, Harvard T.H. Chan School of Public Health, Boston, MA 02115 USA; 16 Escuela Andaluza de Salud Pública (EASP), 18011 Granada, Spain; 17 Instituto de Investigación Biosanitaria ibs.GRANADA, 18012 Granada, Spain; 18 Centro de Investigación Biomédica en Red de Epidemiología y Salud Pública (CIBERESP), 28029 Madrid, Spain; 19 Ministry of Health of the Basque Government, Sub Directorate for Public Health and Addictions of Gipuzkoa, San Sebastian, Spain; 20 BioGipuzkoa (BioDonostia) Health Research Institute, Epidemiology of Chronic and Communicable Diseases Group, San Sebastián, Spain; 21 Department of Epidemiology, Regional Health Council, IMIB-Arrixaca, Murcia University, Murcia, Spain; 22 Instituto de Salud Pública y Laboral de Navarra, 31003 Pamplona, Spain; 23 Navarra Institute for Health Research (IdiSNA), 31008 Pamplona, Spain; 24 Cancer Epidemiology Unit, University of Oxford, Oxford, UK; 25 Department of Epidemiology and Biostatistics, School of Public Health, Imperial College London, London, United Kingdom

**Keywords:** Breast cancer, Cancer epidemiology, Prognostic markers

## Abstract

**Background:**

Inflammation influences tumour progression and cancer prognosis, but its role preceding breast cancer (BC) and its prognostic implications remain inconclusive.

**Methods:**

We studied pre-diagnostic plasma inflammatory biomarkers in 1538 women with BC from the EPIC study. Cox proportional hazards models assessed their relationship with all-cause and BC-specific mortality, adjusting for tumour characteristics and lifestyle factors.

**Results:**

Over a 7-year follow-up after diagnosis, 229 women died, 163 from BC. Elevated IL-6 levels were associated with increased all-cause mortality risk (HR_1-SD_ 1.25, 95% CI 1.07–1.47). Among postmenopausal, IL-6 was associated with higher all-cause (HR_1-SD_ 1.41, 95% CI 1.18–1.69) and BC-specific mortality (HR_1-SD_ 1.31, 95% CI 1.03–1.66), (*P*_Heterogeneity (pre/postmenopausal)_ < 0.05 for both), while IL-10 and TNFα were associated with all-cause mortality only (HR_1-SD_ 1.19, 95% CI 1.02–1.40 and HR_1-SD_ 1.28, 95% CI 1.06–1.56). Among ER+PR+, IL-10 was associated with all-cause and BC-specific mortality (HR_1-SD_ 1.35, 95% CI 1.10–1.65 and HR_1-SD_ 1.42 95% CI 1.08–1.86), while TNF-α was associated with all-cause mortality in HER2- (HR_1-SD_ 1.31, 95% CI 1.07–1.61). An inflammatory score predicted higher all-cause mortality, especially in postmenopausal women (HR_1-SD_ 1.30, 95% CI 1.07–1.58).

**Conclusions:**

Higher pre-diagnosis IL-6 levels suggest poorer long-term survival among BC survivors. In postmenopausal survivors, elevated IL-6, IL-10, and TNFα and inflammatory scores seem to predict all-cause mortality.

## Introduction

Breast cancer (BC) remains a global health concern, standing as the most frequently diagnosed cancer and ranking as the fifth leading cause of cancer-related deaths worldwide [1]. Although breast cancer is a tumour markedly affected by hormone metabolism, especially oestrogens, there is an established relationship between certain modifiable factors and the risk of this cancer. These factors include alcohol consumption, excess adiposity, and the protective effect of physical activity, all of which are characterised by the pathway of chronic and low-grade systemic inflammation [2].

Cancer-associated inflammation plays a significant role in the progression of proliferation and metastasis, stimulating angiogenesis, suppressing antitumor immunity, and ultimately leading to a poor prognosis [3]. However, the role of inflammation preceding BC diagnosis and its prognostic implications remains less clear. Additionally, given the inherent heterogeneity of breast cancer, it has been suggested that inflammation may contribute to poor responsiveness to endocrine therapy, particularly among luminal breast cancers, which account for over 70% of all BC cases [4].

To investigate the relationship between inflammatory status and BC prognosis, several studies have predominantly relied on measurements of C-reactive protein (CRP) in samples collected at different time points with respect to diagnosis [5–8]. These studies have yielded conflicting results, highlighting the need to better understand this relationship, not only with CRP but also with other inflammatory biomarkers. By exploring biomarkers prior to diagnosis, the influence of preceding or “latent” inflammation can be better captured and can provide insight into its impact on BC survival. This would help increase our ability to predict BC outcomes, which may help develop targeted interventions that can improve treatment strategies and ultimately impact survival rates.

In this study, we evaluated the associations between inflammatory biomarkers and survival in women diagnosed with BC. To achieve this, we selected a panel of cytokines, including interleukin (IL)-6, IL-8, IL-10, IL-13, IL-17D, IL-1RA, tumour necrosis factor alpha (TNF-α), Interferon-gamma (IFN-γ), and the adipokines leptin and adiponectin, as well as the acute-phase protein CRP, all measured before BC diagnosis as part of the European Prospective Investigation into Cancer and Nutrition (EPIC). Our aim was to determine whether circulating levels of these inflammatory biomarkers, individually or combined in a composite score, are associated with overall and BC-specific mortality in BC survivors.

## Methods

### Study design and participants

EPIC is a cohort study that recruited more than half a million participants from 10 countries, between 1992 and 2000. The study design and methods of EPIC have been described in detail elsewhere [9]. For the present analyses, data were available from eight EPIC countries: France, Italy, Spain, the United Kingdom (UK), the Netherlands, Germany, Sweden, and Denmark.

Blood samples were obtained following a standard protocol in France, Germany, Italy, the Netherlands, Spain, and the UK. Serum, plasma, erythrocytes, and buffy coat aliquots were stored in liquid nitrogen (−196 °C) in a centralised biobank at the International Agency for Research on Cancer (IARC). In Denmark, blood fractions were stored locally in the vapour phase of liquid nitrogen containers at −150 °C, and in Sweden, they were stored locally in standard freezers at −80 °C [10].

All participants provided written informed consent for data collection and storage, as well as individual follow-up. The EPIC study was approved by the Ethics Committee of the IARC, Lyon, France, as well as the local ethics committee of each study centre. All methods were performed in accordance with the relevant guidelines and regulations.

### Breast cancer cases and outcome assessment

Incident BC cases and vital status were assessed during follow-up based on population cancer registries or national cancer registries and national mortality registries in Denmark, Italy, the Netherlands, Spain, Sweden, and the UK. Cancer cases in France and Germany were identified through cancer and pathology registries, health insurance records, and proactive follow-up by contacting participants or their next-of-kin. BC cases were defined as malignant tumours coded C50.0–50.9 in the International Classification of Diseases for Oncology.

After exclusion of women with prevalent cancer at blood collection and with no follow-up data or no lifestyle information, 327,927 women remained, among whom 13,671 had invasive BC. Exclusions were made if BC cases did not have a blood sample available, where time from recruitment to diagnosis was <2 years or had no information on hormone receptor status (oestrogen receptor (ER), progesterone receptor (PR), and human epidermal growth factor receptor 2 (HER2)). After these exclusions, 1595 BC cases were included for measurement of biomarkers. A total of 57 cases had incomplete information on tumour characteristics or vital status. The final population included 1538 breast cancer cases (Figure S1).

### Inflammatory biomarker assessment

A selection of cytokines (IL-6, IL-8, IL-10, IL-13, IL-17D, IL-1RA, TNF-α, IFN-γ), adipokines (leptin and adiponectin) and CRP were measured on plasma samples in the laboratories of the Nutrition and Metabolism Branch at IARC, by Meso Scale Discovery (a commercially available and highly sensitive immunoassay platform) [10]. No measurements below the lower limit of quantification (LOQ) were observed for leptin, CRP, IL1-RA, IL-8, and IFN-γ. Measurements below the LOQ represented less than 3% for adiponectin and IL-17D, less than 8% for IL-6, and less than 22% for IL-10 and TNFα, and around 80% for IL-13 (Table 1). If biomarker measurements fell below the LOQ, the value was replaced with half the LOQ. Given that a substantial part of IL-13 was below this threshold, a dichotomous variable was used above or below the LOQ.

**Table 1:** Summary statistics of the included inflammatory biomarkers in the analyses (*n* = 1538 breast cancer cases).

Biomarkers	units	*n* < LOQ	%	Geometric mean (95% CI)	Missing values
IL-6	pg/mL	111	7.2	0.43 (0.42–0.45)	4
IL-8	pg/mL	0	0	2.70 (2.61–2.79)	4
IL-10	pg/mL	326	21.2	0.13 (0.13–0.14)	4
IL-13	pg/mL	1234	80.2	0.28 (0.28–0.29)	5
IL-17D	pg/mL	41	2.7	6.67 (6.47–6.87)	5
IL1-RA	pg/mL	0	0	159.46 (155.16–163.88)	5
IFN-γ	pg/mL	0	0	2.98 (2.88–3.09)	4
TNF-α	pg/mL	382	24.8	1.06 (1.03–1.08)	4
CRP	µg/mL	0	0	1.12 (1.05–1.19)	1
Leptin	ng/mL	0	0	9.05 (8.65–9.47)	1
Adiponectin	µg/mL	2	0.1	10.52 (10.25–10.79)	0

*LOQ* limit of quantification.

### Covariates

At recruitment, dietary, lifestyle, reproductive, and medical data were collected using questionnaires, and anthropometric measurements were recorded [9]. Menopausal status at diagnosis was determined by combining the baseline information with the data collected during a second assessment of lifestyle and reproductive factors. Women whose age at diagnosis was 55 years or older were classified as postmenopausal, regardless of the information collected during the initial recruitment phase. If women were initially identified as non-users of hormonal replacement treatment at recruitment but reported ever using hormonal replacement treatment in a subsequent follow-up questionnaire before diagnosis, they were categorised as “ever users” at the time of diagnosis.

Additionally, we utilised the inflammatory score of diet (ISD) [11], a measure gauging the inflammatory potential of diet that ranks individuals based on their consumption of either more pro-inflammatory or more anti-inflammatory diets. We investigated the associations between inflammatory markers and mortality outcomes within two groups: those consuming pro-inflammatory diets and those favouring anti-inflammatory diets. Previous studies in EPIC study showed a positive association between pro-inflammatory diets with risk of BC and, more modestly, risk of overall mortality among BC patients [12, 13].

### Statistical analyses

Log-transformed biomarker concentrations were used in all analyses. To comprehensively assess the association between inflammatory biomarkers and BC survival, we created two composite inflammatory scores. The first score combined all the inflammatory biomarkers (without IL-13), while the second score was constructed without including the adipokines (leptin and adiponectin). The inflammatory scores were derived from log-transformed biomarker concentrations standardised using the mean and SD of our population (*z*-score) of 1538 BC cases; the z-scores of adiponectin were multiplied by −1 to account for its anti-inflammatory effect. These *z*-scores were summed to generate an overall inflammatory score for each BC case.

Baseline characteristics of BC cases were described using frequency and percentages or mean and standard deviation (SD). Geometric means were used to describe biomarker concentrations among BC cases (Table 1). Partial Spearman’s correlations, adjusted for age at the time of blood collection and laboratory batch, were calculated for the biomarkers, and also with ISD and BMI.

We used Cox proportional hazards regression to estimate hazard ratios (HRs) and 95% confidence intervals (CIs) for 1-SD increase in individual inflammatory biomarkers and composite inflammatory scores. IL-13 was treated as a categorical variable based on values above and below LOQ. The outcomes assessed were all-cause mortality and BC-specific mortality. For BC-specific mortality, Fine and Gray subdistribution hazard models were employed, accounting for other causes of death as competing events [14]. Entry time was defined as the date of diagnosis, and exit time was determined by death, emigration, or end of follow-up, with age as the underlying timescale.

All survival models were stratified by country, menopausal status at diagnosis and stage of tumour (metastatic, non-metastatic, unknown), and adjusted for age at diagnosis and laboratory batch (continuous), fasting status, education level, physical activity, body mass index (BMI), alcohol consumption, smoking status and intensity, ever use of hormones for menopause at diagnosis, cancer grade, and tumour receptor status (ER+/−, PR+/−, HER2+/−). The categories used for these covariates are displayed in Table 2. Model assumptions were evaluated with graphs and tests based on the scaled Schoenfeld residuals. Additionally, separate survival analyses were performed according to menopausal status at diagnosis. Further subgroup analyses were performed for the identified associations (IL-6, IL-10, TNFα, and inflammatory scores) according to lifestyle factors, including BMI, physical activity, and anti-inflammatory vs. pro-inflammatory diets. Tumour characteristics, including stage and hormone receptor status, were also considered in subgroup analyses. To assess between-group heterogeneity, Likelihood ratio (LR) tests were used by adding an interaction term in the model between biomarker exposure and the group-defining variable.

**Table 2:** Descriptive statistics of the breast cancer survivors (*N* = 1538) with available inflammatory biomarkers in the EPIC study.

Sociodemographic, lifestyle and reproductive factors	*N*/mean	%/SD
Age at diagnosis (years)	61.4	8.3
Time from blood collection to diagnosis (years)	8.7	2.8
Level of school (*N* unknowns = 29)		
None/Primary	550	35.8
Technical/Professional	379	24.6
Secondary	259	16.8
Longer education	321	20.9
BMI		
Normal weight	762	49.5
Overweight	524	34.1
Obesity	236	15.3
Underweight	16	1.0
Physical activity (*N* unknowns = 8)		
Inactive	343	22.3
Moderately inactive	566	36.8
Moderately active	344	22.4
Active	277	18.0
Smoking status (*N* unknowns = 14)		
Never	772	50.2
Current, 1–15 cig/day	193	12.5
Current, 16–25 cig/day	95	6.18
Current, +26 cig/day	23	1.5
Former, quit ≤10 years	122	7.93
Former, quit ≤11–20 years	114	7.41
Former, quit +20 years	95	6.18
Miscellaneous^a^	113	7.35
Alcohol consumption (g/day)		
Non-drinker	218	14.2
>0–3	416	27.0
>3–12	434	28.2
>12–24	269	17.5
>24	201	13.1
Menopausal status at diagnosis		
Premenopausal	366	23.8
Postmenopausal	1172	76.2
Use of contraceptive pill/hormones for menopause at blood collection		
No	1058	68.8
Yes	480	31.2
Ever use of hormones for menopause at diagnosis (*N* unknowns = 23)		
No	589	50.3
Yes	560	47.3
Tumour characteristics		
Stage of tumour		
Stage I	399	25.9
Stage II	372	24.2
Stage III	93	6.0
Non-metastatic unknown stage	347	22.6
Stage IV	178	11.6
Unknown	149	9.7
Grade of tumour		
Well-differentiated	170	11.1
Moderately differentiated	535	34.8
Poorly differentiated/undifferentiated	353	23.0
Not determined	480	31.2
Oestrogen receptor status		
Negative	300	19.5
Positive	1238	80.5
Progesterone receptor status		
Negative	490	31.9
Positive	1048	68.1
HER2 status		
Negative	1205	78.3
Positive	333	21.7
Triple-negative subtype		
ER−PR−HER2−	112	7.3

^a^Current smoker of cigars, pipes and occasional current smokers, current smokers with missing information on the intensity.

Several sensitivity analyses were performed to assess the robustness of the results. First, we calculated the inflammatory scores without IL-6 to test to what extent this biomarker drove the observed association with the score and analysed its relationship with all-cause mortality, overall as well as according to menopausal status. Second, models were further adjusted by time from blood collection to diagnosis (continuous) and separately analysed by short- and long-term periods (below and above median years). We also repeated the main analyses excluding cases with a short survival time after diagnosis (less than 1 and 2 years) to rule out the potential influence of other factors that could be significant in determining prognosis beyond the biomarkers studied. Cancer grade and hormone receptor status, while strong prognosis factors, have no true confounding effect due to the timing of biomarker measurements, which occurred prior to diagnosis. Considering this, we conducted separate analyses in which these variables were excluded from our models. Finally, we restricted our analyses to non-users of exogenous hormones at blood collection as the use of hormone replacement therapy (HRT) or oral contraceptives may influence inflammatory biomarker levels, apart from breast cancer risk itself, which could confound the associations we intended to examine.

## Results

### Baseline characteristics

The characteristics of the included 1538 BC cases in the EPIC study are summarised in Table 2. After a median follow-up of 7 years from diagnosis, there were 229 deaths, including 163 BC-specific deaths. On average, circulating biomarkers of inflammation were measured 8.7 (Interquartile range: 6.2–10.7) years before BC diagnosis. The average age at BC diagnosis was 61 years, with approximately 75% of cases occurring after menopause. At blood collection, approximately half of the women had a normal BMI, were physically inactive, non-smokers, and consumed alcohol moderately. Early stages (I, II) comprised 50.1% of cases, whereas small proportions had stage III (6%) and metastatic tumours (11.6%). Of all BC cases, 80.5%, 68.1% and 78.3% were ER-positive, PR-positive and HER2-negative, respectively. A total of 158 cases were triple-negative cases (7.3%).

### Correlations between inflammatory biomarkers

**Fig. 1:**
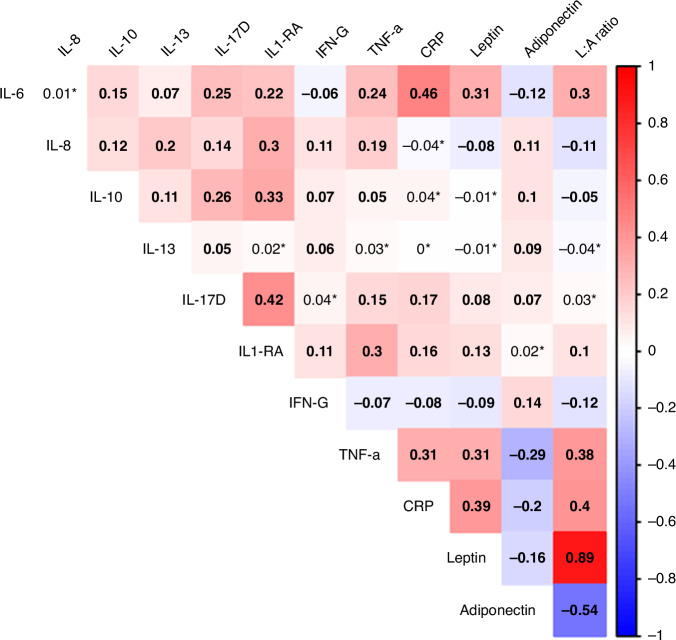
Spearman correlations of log-transformed inflammatory biomarkers adjusted for laboratory batch and age at blood collection. *Asterisks indicate non-statistically significant correlations (*p* > 0.05).

Overall, age- and batch-adjusted Spearman correlations showed positive correlations between biomarkers, except adiponectin (Fig. 1). The highest positive correlation coefficients were observed between IL-6 and CRP (*r* = 0.46), IL-17D and IL1-RA (*r* = 0.42), CRP and leptin-to-adiponectin ratio (L:A ratio) (*r* = 0.40), CRP and leptin (*r* = 0.39), and TNFα and L:A ratio (*r* = 0.38). Adiponectin showed the strongest inverse correlation coefficients, especially with TNFα, CRP, leptin, and IL-6 (r ranged between −0.12 and −0.29). BMI showed positive correlations, especially with leptin (*r* = 0.68) and L:A ratio (*r* = 0.66), CRP (*r* = 0.43), and IL-1RA (*r* = 0.37). Inflammatory diets according to ISD showed weaker positive correlations with IL-17D (*r* = 0.07), IFN-γ (*r* = 0.09), L:A ratio (*r* = 0.13), leptin (*r* = 0.14), CRP (*r* = 0.14), IL-6 (*r* = 0.16).

### Associations between inflammatory biomarkers and BC survival, overall and by menopausal status at diagnosis

**Fig. 2:**
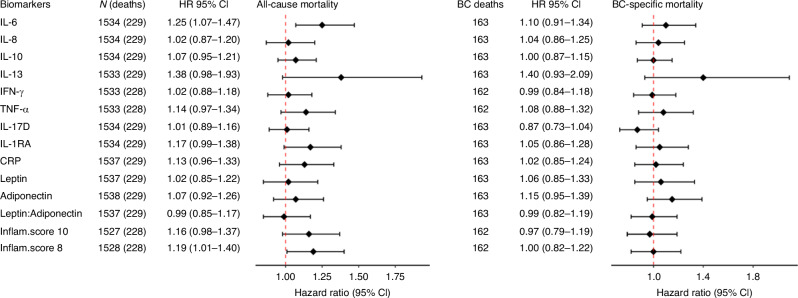
Associations between inflammatory biomarkers (per 1-SD increment) and all-cause and breast cancer-specific mortality among breast cancer cases with available inflammatory biomarkers in EPIC. All individual biomarkers and scores have been assessed on a continuous scale, per 1-SD increase (unless for IL-13, dichotomised based on values above and below the LOQ). Models are stratified by country, menopausal status at diagnosis and stage of tumour (metastatic, non-metastatic, unknown), and adjusted for age at diagnosis, laboratory batch, fasting status at blood collection, education level, physical activity, body mass index, alcohol consumption, smoking status and intensity, ever use of hormones for menopause at diagnosis, cancer grade, and tumour receptor status (ER, PR, HER2). The Inflammatory scores are derived from log-transformed biomarker concentrations standardised using the mean and standard deviation of our population (z-score) of 1538 BC cases; the z-scores for adiponectin were multiplied by −1 to account for its anti-inflammatory effect. These z-scores are summed to generate an overall inflammatory score for each individual. The Inflammatory score-10 is composed of the following biomarkers: IL6, IL8, IL10, IL17D, IL-1RA, IFN-γ, TNFα, CRP, Leptin, and Adiponectin; the Inflammatory score-8 includes the same components as the Inflammatory score-10, except for the adipokines Leptin and Adiponectin. BC breast cancer, HR hazard ratio, CI confidence interval, Inflam.score Inflammatory score, SD standard deviation, IL interleukin, IL-1RA interleukin-1 receptor antagonist, IFN‐γ interferon‐gamma, TNF-α tumour necrosis factor alpha, CRP C-reactive protein.

**Fig. 3:**
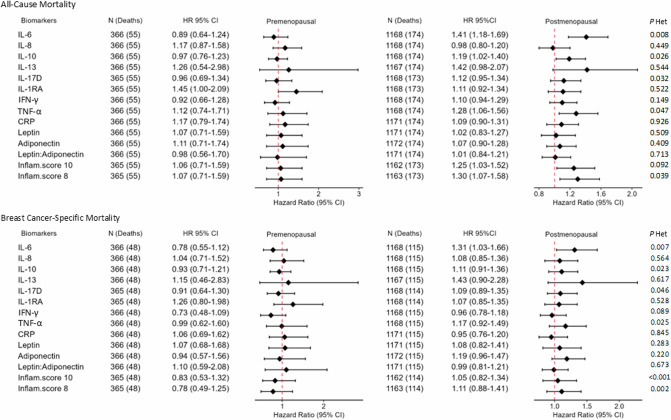
Associations between inflammatory biomarkers (per 1-SD increment) and all-cause and breast cancer-specific mortality according to menopausal status at diagnosis. Individual biomarkers and scores have been assessed on a continuous scale, per 1-SD increase. Models are stratified by country and stage of tumour (metastatic, non-metastatic, unknown), and adjusted for age at diagnosis, laboratory batch, fasting status at blood collection, education level, physical activity, body mass index, alcohol consumption, smoking status and intensity, ever use of hormones for menopause at diagnosis, cancer grade, and tumour receptor status (ER, PR, HER2). The Inflammatory scores are derived from log-transformed biomarker concentrations standardised using the mean and standard deviation of our population (z-score) of 1538 BC cases; the z-scores for adiponectin were multiplied by -1 to account for its anti-inflammatory effect. These z-scores are summed to generate an overall inflammatory score for each individual. The Inflammatory score-10 is composed of the following biomarkers: IL6, IL8, IL10, IL17D, IL-1RA, IFN-γ, TNFα, CRP, Leptin, and Adiponectin; the Inflammatory score-8 includes the same components as the Inflammatory score-10, except for the adipokines Leptin and Adiponectin. BC breast cancer, HR hazard ratio, CI confidence interval, Inflam.score Inflammatory score, SD standard deviation, IL interleukin, TNF-α tumour necrosis factor alpha, Het Heterogeneity.

HRs and 95% CIs for 1-SD increase in individual inflammatory biomarkers and inflammatory scores for all-cause and BC-specific mortality are listed in Fig. 2. Elevated levels of IL-6 before diagnosis were associated with higher all-cause mortality (HR_1-SD_ 1.25, 95% CI 1.07–1.47). The composite inflammatory score without adipokines was also positively associated with all-cause mortality (HR_1-SD_ 1.19, 95% CI 1.01–1.40). No statistically significant association was observed for BC-specific mortality among all BC cases. In analyses by menopausal status at diagnosis (Fig. 3), among postmenopausal women, IL-6 was positively associated with all-cause mortality (HR_1-SD_ 1.41, 95% CI 1.18–1.69; *P*_Het pre/post_ = 0.008) and BC-specific mortality (HR_1-SD_ 1.31, 95% CI 1.03–1.66; *P*_Het pre/post_ = 0.007), while IL-10 and TNF-α were positively associated with all-cause mortality only.

### Associations between inflammatory biomarkers and BC survival by tumour characteristics and BC subtypes

Subgroup analyses for the main associations with all-cause and BC-specific mortality according to tumour characteristics are presented in Table 3. Among cases with non-metastatic tumours (stages I-III), IL-10 showed a positive association with all-cause mortality (HR_1-SD_ 1.32, 95% CI 1.09–1.59). This association was even stronger when restricted to early stages I-II (HR_1-SD_ 1.42, 95% CI 1.16–1.72). Moreover, elevated levels of IL-10 were associated with a higher risk of all-cause mortality in cases with ER+PR+ tumours (HR_1-SD_ 1.35, 95% CI 1.10–1.65). Similarly, IL-10 was positively associated with BC-specific mortality among early stages I-II and ER+PR+ cases. TNF-α was associated with higher all-cause mortality among cases with HER2- tumours, but not in those with HER2+ tumours. All these associations showed statistically significant heterogeneity between subgroups (*P*_Heterogeneity_ <0.05).

**Table 3:** Subgroup analyses according to tumour characteristics for all-cause and breast cancer-specific mortality.

All-cause mortality	*N*	Deaths	IL-6	IL-10	TNF-ɑ	Inflam.score 10	Inflam.score 8
			HR (95% CI)	HR (95% CI)	HR (95% CI)	HR (95% CI)	HR (95% CI)
Stage I, II	771	77	1.06 (0.79–1.42)	**1.42 (1.16–1.72)**	1.27 (0.95–1.69)	1.06 (0.80–1.41)	1.07 (0.81–1.42)
Stage III, IV	271	89	0.99 (0.73–1.34)	1.00 (0.81–1.23)	1.12 (0.82–1.52)	1.13 (0.81–1.57)	1.16 (0.85–1.59)
P-Heterogeneity			0.284	**0.009**	0.468	0.243	0.463
Stage I, II, III	864	100	1.13 (0.88–1.45)	**1.32 (1.09–1.59)**	**1.31 (1.02–1.69)**	1.12 (0.87–1.43)	1.14 (0.89–1.46)
Stage IV	178	66	**1.69 (1.04–2.74)**	1.21 (0.94–1.56)	1.40 (0.91–2.15)	**2.22 (1.30–3.8)**	**2.53 (1.46–4.35)**
P-Heterogeneity			0.481	**0.042**	0.278	0.445	0.650
ER+PR+	1018	117	**1.41 (1.12–1.77)**	**1.35 (1.10–1.65)**	**1.38 (1.08–1.75)**	**1.36 (1.06–1.75)**	**1.37 (1.09–1.74)**
ER−PR−	270	76	1.26 (0.95–1.69)	1.00 (0.79–1.27)	0.97 (0.71–1.31)	1.02 (0.77–1.35)	1.13 (0.85–1.51)
P-Heterogeneity			0.817	**<0.001**	0.067	0.169	0.503
HER2−	1205	159	1.19 (0.98–1.45)	**1.17 (1.00–1.36)**	**1.31 (1.07–1.61)**	1.19 (0.97–1.46)	**1.23 (1.01–1.49)**
HER2+	333	70	1.38 (0.94–2.03)	1.19 (0.88–1.62)	0.93 (0.65–1.34)	1.12 (0.74–1.69)	1.08 (0.73–1.6)
P-Heterogeneity			0.594	0.062	**0.020**	0.131	0.054
Non-triple-negative	1380	188	**1.27 (1.07–1.50)**	**1.14 (1.00–1.30)**	**1.25 (1.05–1.49)**	**1.28 (1.07–1.54)**	**1.25 (1.04–1.49)**
Triple-negative	158	41	1.12 (0.71–1.77)	0.81 (0.57–1.15)	1.26 (0.85–1.88)	0.81 (0.55–1.19)	0.90 (0.61–1.34)
P-Heterogeneity			0.923	0.404	0.432	0.177	0.565

BC-specific mortality	*N*	Deaths	IL-6	IL-10	TNF a	Inflam.score 10	Inflam.score 8
			HR (95% CI)	HR (95% CI)	HR (95% CI)	HR (95% CI)	HR (95% CI)
Stage I, II	771	52	0.88 (0.61–1.26)	**1.39 (1.05–1.84)**	1.03 (0.73–1.45)	0.73 (0.51–1.04)	0.74 (0.52–1.06)
Stage III, IV	271	69	0.77 (0.54–1.11)	0.93 (0.73–1.18)	0.97 (0.66–1.41)	0.96 (0.66–1.4)	0.99 (0.69–1.42)
P-Heterogeneity			0.309	0.056	0.505	0.631	0.982
Stage I, II, III	864	68	0.93 (0.68–1.27)	1.23 (0.94–1.59)	1.12 (0.83–1.50)	0.80 (0.59–1.09)	0.83 (0.61–1.12)
Stage IV	178	53	1.20 (0.70–2.03)	1.21 (0.89–1.63)	1.44 (0.82–2.51)	**2.05 (1.11–3.77)**	**2.74 (1.43–5.27)**
P-Heterogeneity			0.36	0.187	0.227	0.821	0.844
ER+PR+	1018	79	1.16 (0.85–1.56)	**1.42 (1.08–1.86)**	1.28 (0.95–1.71)	1.13 (0.82–1.54)	1.14 (0.84–1.54)
ER−PR−	270	57	1.44 (0.89–2.33)	0.93 (0.71–1.2)	1.04 (0.71–1.52)	0.96 (0.68–1.33)	1.08 (0.76–1.53)
P-Heterogeneity			0.734	**<0.001**	0.211	0.471	0.86
HER2−	1205	107	0.98 (0.77–1.26)	1.12 (0.92–1.35)	1.18 (0.92–1.53)	0.97 (0.76–1.25)	1.01 (0.8–1.29)
HER2+	333	56	1.45 (0.90–2.34)	1.11 (0.78–1.59)	0.86 (0.56–1.33)	0.92 (0.56–1.5)	0.89 (0.56–1.43)
P-Heterogeneity			0.763	0.086	0.157	0.6	0.313
Non-triple-negative	1380	188	1.08 (0.87–1.33)	1.00 (0.82–1.23)	1.08 (0.87–1.32)	1.02 (0.81–1.28)	1.02 (0.82–1.27)
Triple-negative	158	30	1.10 (0.66–1.84)	0.30 (0.39–1.35)	**1.76 (1.06–2.94)**	0.64 (0.40–1.04)	0.90 (0.56–1.47)
P-Heterogeneity			0.948	0.460	0.614	0.231	0.834

Individual biomarkers and scores have been assessed on a continuous scale, per 1 standard deviation (1-SD) increase.

Models stratified by country and menopausal status at diagnosis and adjusted for age at diagnosis, laboratory batch, fasting status at blood collection, education level, physical activity, body mass index, alcohol consumption, smoking status and intensity, ever use of hormones for menopause at diagnosis, cancer grade.

The Inflammatory scores are derived from log-transformed biomarker concentrations standardised using the mean and standard deviation of our population (z-score) of 1538 BC cases; the z-scores for adiponectin were multiplied by −1 to account for its anti-inflammatory effect. These z-scores are summed to generate an overall inflammatory score for each individual. The Inflammatory score-10 is composed of the following biomarkers: IL6, IL8, IL10, IL17D, IL-1RA, IFN-γ, TNFα, CRP, Leptin, and Adiponectin; the Inflammatory score-8 includes the same components as the Inflammatory score-10, except for the adipokines Leptin and Adiponectin.

Bold values indicate statistically significant associations.

*CI* confidence interval, *HR* hazard ratio, *ER* oestrogen receptor, *PR* progesterone receptor, *HER2* human epidermal growth factor receptor 2, *Inflam.score* Inflammatory score, *IL* interleukin, TNF-α tumour necrosis factor alpha.

We performed models among triple-negative and non-triple-negative tumours. Overall, positive associations between IL-6, IL-10, TNF-α, and inflammatory scores with all-cause mortality were evident among cases with non-triple-negative tumours but not among those with triple-negative tumours. (Table 3). However, no statistical evidence of heterogeneity was observed.

There were no statistically significant differences in the associations across subgroups of lifestyle factors, including BMI categories (<25 vs. ≥25 kg/m^2^), physical activity levels (inactive vs. active), and inflammatory diets (anti-inflammatory vs. pro-inflammatory) (Table S1). However, higher IL-6 levels were associated with higher all-cause mortality in cases with a BMI under 25 kg/m^2^, physically inactive, and those consuming anti-inflammatory diets before BC diagnosis. Conversely, IL-10, TNF-α, and the inflammatory score based on cytokines showed positive associations with all-cause mortality among cases with a baseline BMI ≥ 25 kg/m^2^.

### Sensitivity analyses

When the inflammatory scores without IL-6 were analysed, the estimates were attenuated and no longer statistically significant, except among postmenopausal BC cases (Table S2). When models were further adjusted for the time from blood collection to diagnosis (in continuous), the main findings remained consistent (Table S3). Additionally, separate analyses for short and long-time periods from blood draw to diagnosis, using dichotomised timeframes at the median threshold of 8.7 years, were performed (Table S4). The main associations were observed again for IL-6 in both time periods, being stronger for cases with longer periods and for postmenopausal women. Similarly, TNF-α was strongly associated with all-cause mortality among all breast cancer survivors, with higher associations among postmenopausal cases and those with longer periods from blood draw to diagnosis. Despite this, no conclusions can be drawn about differences between short and long periods, as tests for heterogeneity were not statistically significant in all comparisons. The sensitivity analysis excluding BC cases with a survival time of less than 1 or 2 years after diagnosis, showed similar associations for overall mortality, including for IL-6 among all cases, and IL-6, IL-10, TNF-α, and inflammatory scores among postmenopausal cases (Table S5). To account for the potential influence of external hormonal factors, an analysis was performed exclusively among non-users of contraceptive pills or hormone replacement therapy. The results showed stable findings, particularly in relation to IL-6 and the inflammatory score without adipokines (Table S6). Finally, models excluding cancer grade and hormone receptor status variables showed consistency in the results, particularly regarding the association between IL-6 and all-cause mortality (Table S7).

## Discussion

In this prospective cohort study, elevated pre-diagnosis levels of IL-6 were associated with an increased risk of all-cause mortality among BC patients after adjusting for lifestyle factors, including BMI and tumour characteristics. Additionally, a composite score integrating eight cytokines as a measure of generalised inflammatory state was positively associated with all-cause mortality (19% for 1-SD increase in the score). Among postmenopausal BC cases, positive associations were observed between IL-6, TNF-α, IL-10, inflammatory scores and all-cause mortality, and between IL-6 and BC-specific mortality.

Consistent with our results, previous studies have reported an association between elevated levels of IL-6 levels and poor prognosis in BC patients [15–17]. In particular, Esquivel-Velázquez et al. [16] found that higher serum IL-6 levels, but not its expression in breast tissue, were associated with poorer survival and reduced response to endocrine therapy in metastatic breast cancer. Then, Tsoi et al. [18] further demonstrated that IL-6 receptor (IL-6R) expression in BC tissues is associated with tamoxifen resistance and poor survival. Other studies have also described an association between higher IL-6 levels and poor survival in patients with metastatic disease, also influenced by chemotherapy resistance [19–22]. Interestingly, in our study, the positive associations between IL-6 and poor survival were also observed in non-metastatic BC (stage I-III) without significant differences across stages. While a clinical trial with early-stage BC cases (*N* = 1380) reported that high IL-6 expression was associated with improved BC-specific survival [23], this finding was not consistently reported by other studies included in a meta-analysis (*N* = 3224 cases) [17].

The role of IL-10 and TNF-α in BC prognosis has been limitedly investigated in epidemiological studies. Previous reviews, primarily comprising cell line studies, have suggested that TNF-α promotes invasive and malignant behaviour in BC cells, contributing to tumour growth, progression, and metastasis [16, 24]. Furthermore, TNF-α concentration can increase in response to chemotherapy and radiotherapy, leading to therapeutic resistance and potentially promoting BC recurrence [24]. With respect to IL-10, our study showed positive associations with poor prognosis, including all-cause and BC-specific mortality, among non-metastatic cases, and in ER+PR+ and HER2- tumours. This finding partly contradicts previous evidence, where higher levels of IL-10 correlated with metastatic BC [25, 26]. Although there is limited literature available on this specific relationship, one possible hypothesis is that elevated levels of IL-10 in non-metastatic tumours may contribute to the subsequent transition to metastatic disease, ultimately resulting in poorer survival outcomes.

Generally, our subgroup analyses reflected higher positive associations among patients with ER and PR-positive tumours, although not all showed statistically significant heterogeneity when comparing groups. ER is expressed in approximately 75% of breast tumours, and patients with ER+ tumours generally have a better prognosis than those with ER− tumours. Unfortunately, many patients with ER+ tumours do not respond to endocrine therapy and identifying predictive markers could improve their response. A review compiling evidence from clinical, preclinical, and cell-based studies suggests that targeting both oestrogen production and its effects, along with inflammation, may be an effective therapeutic strategy for women with more aggressive, endocrine-resistant ER+ tumours [27].

In previous epidemiological studies, the prognostic value of inflammation for BC survival has often been examined by assessing circulating levels of CRP. However, these studies have assessed CRP at different time points relative to BC diagnosis, which may have contributed to the conflicting results observed among them [5–8, 28–30]. Regarding pre-diagnosis measurements, one study, based on a small sample size, reported an association between higher CRP levels and improved overall survival [30]. Conversely, a larger cohort study observed that higher CRP levels were associated with increased all-cause mortality, though they did account for BMI in their analyses [28]. Moreover, although IL-6 is involved in CRP production [31], its association with BC survival seemed to be independent of BMI. This suggests that IL-6 may have a more direct impact on tumour behaviour or interact with other factors relevant to BC progression, which are not solely influenced by BMI.

Many tumours are triggered by inflammatory responses, which result in the formation of an inflammatory microenvironment around the tumour, promoting favourable conditions for tumour growth, invasion, metastasis, and resistance to chemotherapeutic drugs [16, 32, 33]. The rationale to investigate the role of inflammatory markers in breast cancer progression is further supported by the association of lifestyle factors such as adiposity and physical activity with breast cancer survival, both of which correlate with chronic inflammation (adiposity increases and physical activity decreases inflammation). However, the precise mechanisms through which chronic inflammation affects BC prognosis remain unclear. In addition, elevated levels of IL-6 and TNF-α have been linked to systemic insulin resistance [34], which can potentially contribute to a poorer prognosis in BC patients [35, 36].

Furthermore, while there was no significant heterogeneity across groups of lifestyle factors, some differences in associations according to BMI, physical activity and diet suggest it is worth considering that the impact of these inflammatory biomarkers on BC prognosis may vary depending on individual lifestyle factors.

### Strengths and limitations

Our study benefits from several strengths, primarily the use of a large European prospective cohort study. This enabled us to investigate the associations between pre-diagnostic inflammatory biomarkers and BC prognosis in a large number of BC cases and events (deaths). We were also able to analyse these associations according to menopausal status and hormone receptor status, including triple-negative tumours. In addition, we could account for several potential confounders, including lifestyle factors, such as BMI, physical activity, reproductive factors, and tumour characteristics. The data also allowed us to evaluate the robustness of the associations performing models further adjusted by time from blood collection to diagnosis and, separately, excluding cases with less than 1 to 2 years of survival after diagnosis.

Our study also has a number of limitations. One is the lack of information on treatment, which is a strong determinant influencing prognosis and survival. To address this, we used information on tumour stage, grade of tumour differentiation, and hormone receptor status as potential surrogates for treatment. These factors often determine the therapeutic approach in cancer patients. Second, the measurement of inflammatory biomarkers was performed only once, which makes the interpretation of our results more complex as many additional conditions can occur over time that impact long-term prognosis. Moreover, although most inflammatory markers have shown good reproducibility over time [37], relying on a single measurement may not fully capture potential variations in levels throughout disease progression or in response to other factors. Another limitation is our uncertainty regarding the extent to which circulating levels of inflammatory biomarkers reflect their levels in breast tissue, which may not accurately represent local inflammatory activity. Third, measurements for IL-13 had a large number of values below LOQ, and we had to dichotomise this biomarker into values higher or lower than the LOQ. In addition, we acknowledge that multiple subgroup analyses were performed in this study, emphasising the importance of replicating these results in other studies before deriving firm conclusions. Finally, the extrapolation of our results to other settings, such as low- and middle-income countries and more ethnically diverse populations, will require further studies to replicate these analyses

## Conclusions

This study suggests that IL-6 concentrations before diagnosis may play an important role in long-term prognosis among BC patients, even after adjustment for age at diagnosis, stage of tumour, and BMI. Among postmenopausal BC patients, higher pre-diagnostic IL-6, IL-10, TNF-α and the composite inflammatory scores are related to poor overall survival, and IL-6 also with poor BC-specific survival. These associations require further investigation, including follow-up measurements with a broader panel of inflammatory components.

## Supplementary information

Supplementary Material Clean version

## Data Availability

For information on how to submit an application for gaining access to EPIC data and/or biospecimens, please follow the instructions at https://login.research4life.org/tacsgr0epic_iarc_fr/access/index.php.

## Code availability

All statistical analyses were performed using R 4.2.2 statistical software. All tests were 2-sided, and *P* values less than 0.05 were considered statistically significant. Code can be made available upon reasonable request.
