# Supplementary material for: Prognostic role of pre-diagnostic circulating inflammatory biomarkers in breast cancer survival: evidence from the EPIC cohort study
Source: Br J Cancer. 2024 Sep 28;131(9):1496–505. doi: 10.1038/s41416-024-02858-6 (PMC11519559; doi:10.1038/s41416-024-02858-6)
Supplement: Supplementary file 1 — Supplementary Material Clean version [file 41416_2024_2858_MOESM1_ESM.docx]

**Supplementary Materials**

**Supplementary Methods.**

**Figure S1.** Flow chart of the study population.

**Table S1.** Subgroup analyses according to lifestyle factors for all-cause and breast cancer-specific mortality.

**Table S2.** Models using the inflammatory scores without IL-6 for all-cause and breast cancer-specific mortality.

**Table S3** Survival models further adjusted by time from blood collection to diagnosis.

**Table S4.** Stratified analysis for short and long period from blood collection to the time of diagnosis.

**Table S5.** Sensitivity analyses excluding breast cancer cases with survival time less than 1 and 2 years after diagnosis.

**Table S6.** Sensitivity analyses excluding breast cancer cases with external hormones use (at diagnosis) including contraceptive pills and hormone replacement treatment for menopause.

**Table S7.** Sensitivity analyses excluding cancer grade, and hormone receptor status from model of all-cause mortality.

**Supplementary Methods**

**Study Population**

The European Prospective Investigation into Cancer and Nutrition (EPIC) is a cohort study that recruited more than half a million of participants from 10 countries, between 1992 and 2000. The study design and methods of EPIC have been described in detail elsewhere (1). The EPIC study was approved by the Ethics Committee of the International Agency for Research on Cancer (IARC), Lyon, France, as well as the local ethics committee of each study centre.

Blood samples were obtained following a standard protocol in France, Germany, Italy, the Netherlands, Spain, and the UK. Serum, plasma, erythrocytes, and buffy coat aliquots were stored in liquid nitrogen (−196 °C) in a centralized biobank at the International Agency for Research on Cancer (IARC). In Denmark, blood fractions were stored locally in the vapour phase of liquid nitrogen containers at −150 °C, and in Sweden, they were stored locally in standard freezers at −80 °C (2).

**Inclusion criteria: Ascertainment of breast cancer cases**

After exclusion of women with prevalent cancer at blood collection and with no follow-up data or no lifestyle information, 327,927 women remained, among whom 13,671 had invasive breast cancer (BC).

Exclusions were made if BC cases did not have a blood sample available, where time from recruitment to diagnosis was <2 years or had no information on hormone receptor status (oestrogen receptor (ER), progesterone receptor (PR), and human epidermal growth factor receptor 2 (HER2). After these exclusions, 1595 BC cases were included for measurement of biomarkers. A total of 57 cases had incomplete information on tumour characteristics or vital status. The final population included 1538 BC cases (Figure S1).

**Inflammatory biomarker assessment**

A selection of cytokines (IL-6, IL-8, IL-10, IL-13, IL-17D, IL-1RA, TNF-α, IFN-γ), adipokines (leptin and adiponectin), and CRP were measured on plasma samples. in the laboratories of the Nutrition and Metabolism Branch at IARC, by Meso Scale Discovery (a commercially available and highly sensitive immunoassay platform) (2). No measurements below the lower limit of quantification (LOQ) were observed for leptin, CRP, IL1-RA, IL-8, and IFN-γ.

**Covariates**

At recruitment, dietary, lifestyle, reproductive, and medical data were collected using questionnaires, and anthropometric measurements were recorded (1). Menopausal status at diagnosis was determined by combining baseline information with data from a second assessment of lifestyle and reproductive factors. Women whose age at diagnosis was 55 years or older were classified as postmenopausal, regardless of the information collected during the initial recruitment phase. If women were initially identified as non-users of hormonal replacement treatment at recruitment but reported ever using hormonal replacement treatment in a subsequent follow-up questionnaire before diagnosis, they were categorized as "ever users" at the time of diagnosis.

Additionally, we utilized the inflammatory score of diet (ISD) (3), a measure of the inflammatory potential of diet ranking individuals based on their consumption of pro-inflammatory or anti-inflammatory diets. This allowed us to explore associations within these two groups. Previous studies with EPIC data showed a positive association between pro-inflammatory diets and risk of BC as well as a more modest association with overall mortality among BC patients (4,5).

**Statistical analyses**

Cox regression models were stratified for menopausal status at diagnosis (pre or postmenopausal) and stage of tumour (metastatic, non-metastatic, unknown), and adjusted for age at diagnosis (5-years categories), laboratory batch (continuous), fasting status at blood collection (yes, no, in between), education level (no formal education, primary school, secondary school, technical or professional training, university, and not specified), physical activity (inactive, moderately inactive, moderately active, active, unknown), body mass index (<18.5, 18.5<25, 25<30, ≥30), alcohol consumption (non-drinker, 0 to < 3 g/day, 3 to < 12 g/day, 12 to < 24 g/day, ≥ 24 g/day, unknown), smoking status and intensity (never smokers, current smokers 1–15, 16–25, and > 25 cigarettes/day, former quit ≤ 10, 11–20, and > 20 years before recruitment, current smoker of cigars, pipes and occasional current smokers, current smokers with unknown intensity, and not specified), ever use of hormones for menopause at diagnosis (yes, no, unknown), grade of tumour (well differentiated, moderately differentiated, poorly differentiated or undifferentiated, not determined), and tumour receptor status (positive, negative) for ER, PR, HER2.

**Figure S1.** Flow chart of the study population.

Final sample included **1538 cases** (women)

**Exclusion of cases with unknown hormone receptor status**

19

**Exclusion of cases with no information of tumour morphology, check date of vital status, date at diagnosis or inconsistent time survival**

38

**327 927 EPIC women**

**13 671 invasive cases**

**Exclusion of cases without blood samples**

4048

**Exclusion of cases with a follow-up time to diagnosis < 2 years**

1020

**Exclusion of cases diagnosed after 31/12/2012**

43

**Exclusion of cases with unavailable receptor status**

5 525

3035 invasive cases available for random sampling

Among them, **1595** invasive breast cancer cases with biomarker measurements

**Table S1.** Subgroup analyses according to lifestyle factors for all-cause and breast cancer-specific mortality.

| **All-cause mortality** | | N | Deaths | IL-6 | IL-10 | TNF-α | Inflam.score 10 | Inflam.score 8 |
| --- | --- | --- | --- | --- | --- | --- | --- | --- |
|  |  |  |  | HR (95% CI) | HR (95% CI) | HR (95% CI) | HR (95% CI) | HR (95% CI) |
|  | BMI <25 | 778 | 103 | **1.38 (1.05-1.82)** | 1.05 (0.86-1.27) | 1.17 (0.89-1.55) | 1.23 (0.91-1.67) | 1.24 (0.93-1.66) |
|  | BMI >=25 | 760 | 126 | 1.22 (0.99-1.5) | **1.21 (1.01-1.46)** | **1.28 (1.02-1.60)** | 1.22 (0.98-1.51) | **1.27 (1.03-1.58)** |
| P-Heterogeneity^1^ | |  |  | 0.703 | 0.227 | 0.497 | 0.705 | 0.443 |
|  | PA Inactive | 909 | 140 | **1.21 (1.00-1.47)** | 1.1 (0.91-1.34) | 1.19 (0.96-1.47) | 1.23 (0.99-1.53) | 1.21 (0.98-1.51) |
|  | PA Active | 621 | 88 | 1.08 (0.78-1.5) | 1.12 (0.89-1.42) | 1.01 (0.73-1.40) | 0.89 (0.63-1.24) | 0.98 (0.70-1.37) |
| P-Heterogeneity^1^ | |  |  | 0.804 | 0.152 | 0.174 | 0.036 | 0.154 |
|  | ISD Antiinflammatory | 385 | 58 | **1.78 (1.11-2.85)** | 0.86 (0.61-1.2) | 1.01 (0.68-1.49) | 0.94 (0.60-1.45) | 0.87 (0.57-1.34) |
|  | ISD Proinflammatory | 386 | 66 | 0.83 (0.58-1.19) | 1.17 (0.93-1.48) | **1.48 (1.01-2.19)** | 1.31 (0.91-1.88) | 1.32 (0.93-1.88) |
| P-Heterogeneity^1^ | |  |  | 0.512 | 0.624 | 0.292 | 0.077 | 0.059 |
| **BC-Specific mortality** | | N | Deaths | IL-6 | IL-10 | TNF-α | Biomarker score 10 | Biomarker score 8 |
|  |  |  |  | HR (95% CI) | HR (95% CI) | HR (95% CI) | HR (95% CI) | HR (95% CI) |
|  | BMI <25 | 778 | 73 | 1.16 (0.82-1.65) | 0.98 (0.78-1.23) | 1.12 (0.8-1.56) | 0.98 (0.67-1.43) | 0.98 (0.68-1.42) |
|  | BMI >=25 | 760 | 90 | 1.15 (0.89-1.48) | 1.11 (0.89-1.4) | 1.16 (0.87-1.54) | 1.08 (0.84-1.4) | 1.15 (0.89-1.49) |
| P-Heterogeneity^1^ | |  |  | 0.307 | 0.197 | 0.479 | 0.351 | 0.202 |
|  | PA Inactive | 909 | 98 | 1.11 (0.88-1.42) | 1.1 (0.86-1.41) | 1.1 (0.85-1.42) | 1.01 (0.77-1.31) | 1.03 (0.8-1.33) |
|  | PA Active | 621 | 64 | 1.03 (0.77-1.38) | 0.98 (0.8-1.21) | 0.95 (0.67-1.36) | 0.81 (0.6-1.1) | 0.9 (0.65-1.24) |
| P-Heterogeneity^1^ | |  |  | 0.89 | 0.158 | 0.22 | 0.288 | 0.424 |
|  | ISD Antiinflammatory | 385 | 41 | 1.41 (0.81-2.48) | 0.77 (0.49-1.23) | 1.08 (0.58-2) | 0.94 (0.51-1.72) | 0.82 (0.45-1.51) |
|  | ISD Proinflammatory | 386 | 50 | 0.69 (0.44-1.08) | 0.97 (0.7-1.34) | 1.23 (0.77-1.97) | 1.13 (0.72-1.77) | 1.07 (0.7-1.64) |
| P-Heterogeneity^1^ | |  |  | 0.804 | 0.801 | 0.631 | 0.081 | 0.086 |

Abbreviations: HR hazard ratio; CI confidence interval; Inflam.score Inflammatory score; IL interleukin; TNF-α tumor necrosis factor alpha; Inflam.score 10 = Inflammatory score including leptin and adiponectin; Inflam.score 8 excludes leptin and adiponectin BC breast cancer.

Inflammatory scores have been assessed on a continuous scale, per 1 standard deviation increase.

^1^P-value of the heterogeneity test using a likelihood ratio test after including an interaction term between the subgroup variable and each of the exposures in the model.

**Table S2.** Models using the inflammatory scores without IL-6 for all-cause and breast cancer-specific mortality.

| **All-cause mortality** | All BC survivors | | Premenopausal BC | | Postmenopausal BC | | P-Het^1^ |
| --- | --- | --- | --- | --- | --- | --- | --- |
|  | N (deaths) | HR (95% CI) | N (deaths) | HR (95% CI) | N (deaths) | HR (95% CI) |  |
| Inflam.score 10 | 1527 (228) | 1.11 (0.94-1.32) | 365 (55) | 1.12 (0.74-1.69) | 1162 (173) | 1.18 (0.97-1.43) | 0.198 |
| Inflam.score 8 | 1528 (228) | 1.14 (0.97-1.34) | 365 (55) | 1.13 (0.75-1.70) | 1163 (173) | **1.22 (1.01-1.47)** | 0.105 |
| **BC-specific mortality** | All BC survivors | | Premenopausal BC | | Postmenopausal BC | | P-Het |
|  | N (deaths) | HR (95% CI) | N (deaths) | HR (95% CI) | N (deaths) | HR (95% CI) |  |
| Inflam.score 10 | 1527 (162) | 0.94 (0.77-1.15) | 365 (48) | 0.89 (0.55-1.43) | 1162 (114) | 0.99 (0.78-1.25) | 0.069 |
| Inflam.score 8 | 1528 (162) | 0.98 (0.80-1.18) | 365 (48) | 0.83 (0.52-1.35) | 1163 (114) | 1.05 (0.83-1.32) | **0.020** |

Abbreviations: HR hazard ratio; CI confidence interval; Inflam.score Inflammatory score; BC breast cancer; Inflam.score 10 = Inflammatory score including leptin and adiponectin; Inflam.score 8 excludes leptin and adiponectin BC breast cancer.

Inflammatory scores have been assessed on a continuous scale, per 1 standard deviation increase.

^1^P-value of the heterogeneity test using a likelihood ratio test after including an interaction term between menopausal status and each of the exposures in the model.

**Table S3.** Survival models further adjusted by time from blood collection to diagnosis.

| Overall mortality | All BC survivors^1^ | | Premenopausal^2^ BC | | Postmenopausal^2^ BC | | P-Het^3^ |
| --- | --- | --- | --- | --- | --- | --- | --- |
|  | N (deaths) | HR | N (deaths) | HR | N (deaths) | HR |  |
| IL-6 | 1534 (229) | **1.25 (1.07-1.47)** | 366 (55) | 0.89 (0.64-1.24) | 1168 (174) | **1.42 (1.18-1.70)** | **0.007** |
| IL-10 | 1534 (229) | 1.07 (0.95-1.21) | 366 (55) | 0.97 (0.76-1.23) | 1168 (174) | **1.19 (1.02-1.40)** | **0.026** |
| TNF-α | 1534 (229) | 1.17 (0.99-1.38) | 366 (55) | 1.13 (0.74-1.72) | 1168 (174) | **1.28 (1.06-1.56)** | **0.048** |
| Inflam.score 10 | 1527 (228) | 1.16 (0.98-1.37) | 365 (55) | 1.06 (0.70-1.60) | 1162 (173) | **1.25 (1.03-1.52)** | 0.093 |
| Inflam.score 8 | 1528 (228) | **1.19 (1.01-1.40)** | 365 (55) | 1.07 (0.71-1.60) | 1163 (173) | **1.30 (1.07-1.57)** | **0.040** |
| BC-specific mortality | All BC survivors^1^ | | Premenopausal^2^ BC | | Postmenopausal^2^ BC | | P-Het |
|  | N (deaths) | HR | N (deaths) | HR | N (deaths) | HR |  |
| IL-6 | 1534 (163) | 1.10 (0.91-1.34) | 366 (48) | 0.78 (0.54-1.12) | 1168 (115) | **1.31 (1.03-1.66)** | **0.007** |
| IL-10 | 1534 (163) | 1.00 (0.87-1.15) | 366 (48) | 0.93 (0.71-1.21) | 1168 (115) | 1.11 (0.91-1.36) | **0.023** |
| TNF-α | 1534 (163) | 1.05 (0.86-1.28) | 366 (48) | 0.99 (0.61-1.60) | 1168 (115) | 1.17 (0.92-1.49) | **0.025** |
| Inflam.score 10 | 1527 (162) | 0.97 (0.79-1.19) | 365 (48) | 0.83 (0.52-1.32) | 1162 (114) | 1.05 (0.82-1.34) | **0.028** |
| Inflam.score 8 | 1528 (162) | 1.00 (0.82-1.24) | 365 (48) | 0.78 (0.49-1.25) | 1163 (114) | 1.13 (0.88-1.41) | **0.006** |

Abbreviations: HR hazard ratio; CI confidence interval; IL interleukin; TNF-α tumor necrosis factor alpha; Inflam.score 10 = Inflammatory score including leptin and adiponectin; Inflam.score 8 excludes leptin and adiponectin BC breast cancer;

^1^Models are stratified by country, menopausal status at diagnosis and stage of tumour (metastatic, non-metastatic, unknown), and adjusted for age at diagnosis, laboratory batch, fasting status at blood collection, education level, physical activity, body mass index, alcohol consumption, smoking status and intensity, ever use of hormones for menopause at diagnosis, cancer grade, and tumour receptor status (ER, PR, HER2).

The variable time from blood collection to diagnosis has been added into the models in continuous.

^2^Same multivariable models without stratification for menopausal status at diagnosis.

^3^P-value of the heterogeneity test using a likelihood ratio test after including an interaction term between menopausal status and each of the exposures in the model.

**Table S4.** Stratified analysis for short and long period from blood collection to the time of diagnosis.

|  | Time from blood draw to diagnosis < 8.7 years | | | | Time from blood draw to diagnosis ≥ 8.7 years | | | | P-Heterogeneity | |
| --- | --- | --- | --- | --- | --- | --- | --- | --- | --- | --- |
|  | All BC survivors | | Postmenopausal BC | | All BC survivors | | Postmenopausal BC | | pvalue^1^ | pvalue^2^ |
| Overall mortality | N (deaths) | HR (95% CI) | N (deaths) | HR (95% CI) | N (deaths) | HR (95% CI) | N (deaths) | HR (95% CI) |  |  |
| IL-6 | 773 (140) | **1.26 (1.02-1.56)** | 543 (98) | **1.55 (1.20-2.01)** | 761 (89) | **1.53 (1.14-2.03)** | 625 (76) | **1.62 (1.18-2.22)** | 0.108 | 0.351 |
| IL-10 | 773 (140) | 1.01 (0.86-1.18) | 543 (98) | 1.21 (0.95-1.54) | 761 (89) | 1.15 (0.91-1.45) | 625 (76) | 1.19 (0.92-1.54) | 0.157 | 0.858 |
| TNF-α | 773 (140) | 1.01 (0.81-1.27) | 543 (98) | 1.07 (0.80-1.42) | 761 (89) | **1.34 (1.02-1.76)** | 625 (76) | **1.46 (1.08-1.99)** | 0.130 | 0.092 |
| Inflam.score 10 | 769 (139) | 1.10 (0.87-1.37) | 539 (97) | 1.17 (0.88-1.54) | 758 (89) | 1.17 (0.88-1.57) | 623 (76) | 1.27 (0.92-1.76) | 0.455 | 0.246 |
| Inflam.score 8 | 769 (139) | 1.13 (0.91-1.40) | 539 (97) | 1.22 (0.93-1.60) | 759 (89) | 1.20 (0.90-1.59) | 624 (76) | 1.29 (0.94-1.78) | 0.564 | 0.401 |
| BC-specific mortality |  |  |  |  |  |  |  |  |  |  |
| IL-6 | 773 (103) | 1.19 (0.92-1.53) | 543 (67) | **1.68 (1.20-2.33)** | 761 (60) | 1.11 (0.75-1.66) | 625 (48) | 1.20 (0.74-1.95) | 0.539 | 0.796 |
| IL-10 | 773 (103) | 1.00 (0.83-1.19) | 543 (67) | 1.25 (0.94-1.66) | 761 (60) | 0.92 (0.66-1.28) | 625 (48) | 0.97 (0.64-1.47) | 0.749 | 0.263 |
| TNF-α | 773 (103) | 1.01 (0.77-1.33) | 543 (67) | 1.20 (0.84-1.72) | 761 (60) | 1.04 (0.74-1.46) | 625 (48) | 1.16 (0.80-1.70) | 0.735 | 0.888 |
| Inflam.score 10 | 769 (102) | 1.01 (0.77-1.33) | 539 (66) | 1.14 (0.81-1.60) | 758 (60) | 0.79 (0.54-1.15) | 623 (48) | 0.91 (0.59-1.42) | 0.970 | 0.786 |
| Inflam.score 8 | 769 (102) | 1.06 (0.82-1.38) | 539 (66) | 1.29 (0.92-1.81) | 759 (60) | 0.78 (0.54-1.13) | 624 (48) | 0.89 (0.58-1.36) | 0.668 | 0.604 |
| Abbreviations: HR hazard ratio; CI confidence interval; IL interleukin; TNF-α tumor necrosis factor alpha; Inflam.score 10 = Inflammatory score including leptin and adiponectin; Inflam.score 8 excludes leptin and adiponectin; BC breast cancer. | | | | | | | | | | |
| pvalue^1^ (among all cases) represents the heterogeneity test between two multivariable models adjusted for time from blood draw to diagnosis, one of which includes the interaction term between the dichotomous variable (below/above 8.7 years) and the exposure. | | | | | | | | | | |
| p-value2 (among postmenopausal cases) represents the heterogeneity test between two multivariable models adjusted for time from blood draw to diagnosis, one of which includes the interaction term between the dichotomous variable (below/above 8.7 years) and the exposure.  Associations among premenopausal cases could not be performed due to the small number of events in each strata of periods of time (12 to 13 in overall and breast cancer-specific mortality, respectively). | | | | | | | | | | |

| **Excluded cases survival < 1year** | | All BC survivors | | | | | Premenopausal BC | | | | | Postmenopausal BC | | | | | P-Het |
| --- | --- | --- | --- | --- | --- | --- | --- | --- | --- | --- | --- | --- | --- | --- | --- | --- | --- |
| **All-cause mortality** | | N | Deaths | HR (95% CI) | | | N | Deaths | HR (95% CI) | | | N | Deaths | HR (95% CI) | | |  |
|  | IL-6 | 1492 | 205 | **1.24 (1.05-1.47)** | | | 356 | 47 | 0.86 (0.60-1.24) | | | 1136 | 158 | **1.41 (1.17-1.71)** | | | **0.011** |
|  | IL-10 | 1492 | 205 | 1.08 (0.95-1.23) | | | 356 | 47 | 1.03 (0.80-1.32) | | | 1136 | 158 | **1.20 (1.01-1.42)** | | | 0.065 |
|  | TNF-α | 1492 | 205 | **1.20 (1.01-1.43)** | | | 356 | 47 | 1.18 (0.76-1.82) | | | 1136 | 158 | **1.33 (1.08-1.62)** | | | 0.075 |
|  | Inflam.score 10 | 1485 | 204 | 1.13 (0.95-1.35) | | | 355 | 47 | 0.99 (0.65-1.50) | | | 1130 | 157 | **1.24 (1.01-1.53)** | | | 0.082 |
|  | Inflam.score 8 | 1486 | 204 | 1.18 (0.99-1.40) | | | 355 | 47 | 1.02 (0.67-1.55) | | | 1131 | 157 | **1.31 (1.07-1.60)** | | | **0.044** |
| **BC-specific mortality** | |  | | | | | |  |  |  |  |  |  |  |  |  |  |
|  | IL-6 | 1492 | 147 | 1.08 (0.88-1.33) | | | 356 | 41 | 0.71 (0.47-1.06) | | | 1136 | 106 | **1.32 (1.03-1.68)** | | | **0.006** |
|  | IL-10 | 1492 | 147 | 1.02 (0.88-1.19) | | | 356 | 41 | 0.99 (0.75-1.30) | | | 1136 | 106 | 1.14 (0.92-1.40) | | | 0.051 |
|  | TNF-α | 1492 | 147 | 1.09 (0.89-1.34) | | | 356 | 41 | 1.00 (0.61-1.62) | | | 1136 | 106 | 1.22 (0.95-1.57) | | | **0.046** |
|  | Inflam.score 10 | 1485 | 146 | 0.96 (0.78-1.19) | | | 355 | 41 | 0.75 (0.46-1.22) | | | 1130 | 105 | 1.07 (0.84-1.38) | | | **0.017** |
|  | Inflam.score 8 | 1486 | 146 | 1.00 (0.81-1.23) | | | 355 | 41 | 0.72 (0.44-1.19) | | | 1131 | 105 | 1.13 (0.89-1.45) | | | **0.005** |
| **Excluded cases survival < 2 year** | | All BC survivors | | | | | Premenopausal BC | | | | | Postmenopausal BC | | | | | P-Het |
| **All-cause mortality** | | N | Deaths | HR (95% CI) | | |  | Deaths | HR | lowCI | upCI | N | Deaths | HR | lowCI | upCI |  |
|  | IL-6 | 1446 | 176 | **1.23 (1.03-1.47)** | | | 344 | 40 | 0.90 (0.60-1.34) | | | 1102 | 136 | **1.42 (1.16-1.73)** | | | **0.006** |
|  | IL-10 | 1446 | 176 | 1.09 (0.96-1.25) | | | 344 | 40 | 1.04 (0.78-1.39) | | | 1102 | 136 | **1.22 (1.02-1.46)** | | | 0.068 |
|  | TNF-α | 1446 | 176 | 1.13 (0.94-1.36) | | | 344 | 40 | 1.16 (0.72-1.85) | | | 1102 | 136 | **1.26 (1.01-1.57)** | | | 0.109 |
|  | Inflam.score 10 | 1439 | 175 | 1.10 (0.91-1.33) | | | 343 | 40 | 1.08 (0.69-1.70) | | | 1096 | 135 | 1.19 (0.96-1.49) | | | 0.19 |
|  | Inflam.score 8 | 1440 | 175 | 1.14 (0.95-1.38) | | | 343 | 40 | 1.09 (0.69-1.71) | | | 1097 | 135 | **1.26 (1.01-1.56)** | | | 0.102 |
| **BC-specific mortality** | |  |  |  |  |  |  |  |  |  |  |  |  |  |  |  |  |
|  | IL-6 | 1446 | 124 | 1.12 (0.89-1.39) | | | 344 | 35 | 0.75 (0.48-1.17) | | | 1102 | 89 | **1.40 (1.08-1.81)** | | | **0.003** |
|  | IL-10 | 1446 | 124 | 1.07 (0.92-1.25) | | | 344 | 35 | 1.02 (0.75-1.37) | | | 1102 | 89 | 1.20 (0.96-1.50) | | | 0.07 |
|  | TNF-α | 1446 | 124 | 1.06 (0.85-1.32) | | | 344 | 35 | 0.96 (0.56-1.62) | | | 1102 | 89 | 1.20 (0.92-1.57) | | | 0.092 |
|  | Inflam.score 10 | 1439 | 123 | 0.96 (0.76-1.20) | | | 343 | 35 | 0.85 (0.51-1.42) | | | 1096 | 88 | 1.06 (0.80-1.39) | | | 0.075 |
|  | Inflam.score 8 | 1440 | 123 | 1.00 (0.80-1.25) | | | 343 | 35 | 0.82 (0.48-1.37) | | | 1097 | 88 | 1.14 (0.87-1.49) | | | **0.026** |

**Table S5.** Sensitivity analyses excluding breast cancer cases with survival time less than 2 years after diagnosis.

Abbreviations: HR hazard ratio; CI confidence interval; IL interleukin; TNF-α tumor necrosis factor alpha; Inflam.score 10 = Inflammatory score including leptin and adiponectin; Inflam.score 8 excludes leptin and adiponectin; BC breast cancer.

P-Het = P-value of the heterogeneity test using a likelihood ratio test after including an interaction term between menopausal status and each of the exposures in the model.

**Table S6.** Sensitivity analyses excluding breast cancer cases with external hormones use (at diagnosis) including contraceptive pills and hormone replacement treatment for menopause.

| **All-cause mortality** | All BC survivors | | Premenopausal BC | | Postmenopausal BC | | P-Het |
| --- | --- | --- | --- | --- | --- | --- | --- |
|  | N (deaths) | HR (95% CI) | N (deaths) | HR (95% CI) | N (deaths) | HR (95% CI) |  |
| IL-6 | 1085 (169) | **1.28 (1.06-1.53)** | 308 (49) | 0.90 (0.63-1.29) | 777 (120) | **1.46 (1.17-1.81)** | **0.028** |
| IL-10 | 1085 (169) | 1.07 (0.93-1.24) | 308 (49) | 0.95 (0.72-1.27) | 777 (120) | 1.20 (0.99-1.46) | **0.049** |
| TNF-α | 1085 (169) | 1.18 (0.96-1.45) | 308 (49) | 1.16 (0.74-1.82) | 777 (120) | 1.26 (0.98-1.62) | 0.245 |
| Inflam.score 10 | 1079 (168) | 1.20 (0.97-1.48) | 307 (49) | 1.09 (0.71-1.69) | 772 (119) | 1.25 (0.97-1.62) | 0.387 |
| Inflam.score 8 | 1080 (168) | **1.24 (1.01-1.52)** | 307 (49) | 1.08 (0.70-1.66) | 773 (119) | **1.34 (1.04-1.72)** | 0.163 |
| **BC-specific mortality** | All BC survivors | | Premenopausal BC | | Postmenopausal BC | | pvalue |
|  | N (deaths) | HR (95% CI) | N (deaths) | HR (95% CI) | N (deaths) | HR (95% CI) |  |
| IL-6 | 1085 (124) | 1.12 (0.90-1.39) | 308 (42) | 0.75 (0.50-1.14) | 777 (82) | **1.34 (1.02-1.76)** | **0.019** |
| IL-10 | 1085 (124) | 0.99 (0.84-1.17) | 308 (42) | 0.90 (0.66-1.24) | 777 (82) | 1.10 (0.87-1.40) | **0.072** |
| TNF-α | 1085 (124) | 1.02 (0.80-1.30) | 308 (42) | 1.03 (0.62-1.71) | 777 (82) | 1.10 (0.80-1.50) | 0.242 |
| Inflam.score 10 | 1079 (123) | 0.98 (0.76-1.25) | 307 (42) | 0.81 (0.49-1.35) | 772 (81) | 1.03 (0.75-1.40) | 0.158 |
| Inflam.score 8 | 1080 (123) | 0.98 (0.77-1.25) | 307 (42) | 0.75 (0.45-1.26) | 773 (81) | 1.08 (0.80-1.46) | 0.066 |

Abbreviations: HR hazard ratio; CI confidence interval; IL interleukin; TNF-α tumor necrosis factor alpha; Inflam.score 10 = Inflammatory score including leptin and adiponectin; Inflam.score 8 excludes leptin and adiponectin; BC breast cancer.

P-Het = P-value of the heterogeneity test using a likelihood ratio test after including an interaction term between menopausal status and each of the exposures in the model.

**Table S7.** Sensitivity analyses excluding cancer grade, and hormone receptor status from model of all-cause mortality.

| All-cause mortality | All BC survivors^1^ | |
| --- | --- | --- |
|  | N (Deaths) | HR 95% CI |
| IL-6 | 1534 (229) | 1.19 (1.02-1.39) |
| IL-8 | 1534 (229) | 0.99 (0.84-1.15) |
| IL-10 | 1534 (229) | 1.11 (0.99-1.25) |
| IL-13 | 1533 (229) | 1.38 (0.99-1.92) |
| IL-17D | 1533 (228) | 1.03 (0.89-1.18) |
| IL-1RA | 1533 (228) | 1.09 (0.93-1.28) |
| IFN-γ | 1534 (229) | 1.01 (0.89-1.16) |
| TNF-ɑ | 1534 (229) | 1.18 (1.00-1.38) |
| CRP | 1537 (229) | 1.11 (0.95-1.30) |
| Leptin | 1537 (229) | 1.05 (0.88-1.27) |
| Adiponectin | 1538 (229) | 1.03 (0.88-1.20) |
| Leptin:Adiponectin ratio | 1537 (229) | 1.01 (0.84-1.21) |
| Inflam.score 10 | 1527 (228) | 1.16 (0.98-1.36) |
| Inflam.score 8 | 1528 (228) | 1.16 (0.99-1.36) |
| Abbreviations: HR hazard ratio; CI confidence interval; IL interleukin; IL-1RA interleukin-1 receptor antagonist; IFN‐γ Interferon‐gamma; TNF-α tumor necrosis factor alpha; CRP C-reactive protein; Inflam.score 10 = Inflammatory score including leptin and adiponectin; Inflam.score 8 excludes leptin and adiponectin; BC breast cancer.  ^1^HRs were estimated per 1 standard deviation increase in log-transformed biomarkers concentrations from multivariable Cox regression models (unless for IL-13, dichotomized based on values above and below the lower limit of quantification). | | |
| Multivariable Cox models adjusted for educational level, body mass index, physical activity, alcohol consumption, age at diagnosis, fasting status, batch effect, smoking status and intensity, ever use of menopausal hormonal therapy, and stratified by stage of tumour, country and menopausal status at diagnosis. | | |

1. Riboli E, Hunt KJ, Slimani N, Ferrari P, Norat T, Fahey M, et al. European Prospective Investigation into Cancer and Nutrition (EPIC): study populations and data collection. Public Health Nutr. desembre 2002;5(6B):1113-24.

2. Cairat M, Rinaldi S, Navionis AS, Romieu I, Biessy C, Viallon V, et al. Circulating inflammatory biomarkers, adipokines and breast cancer risk—a case-control study nested within the EPIC cohort. BMC Medicine. 18 abril 2022;20(1):118.

3. Agudo A, Cayssials V, Bonet C, Tjønneland A, Overvad K, Boutron-Ruault MC, et al. Inflammatory potential of the diet and risk of gastric cancer in the European Prospective Investigation into Cancer and Nutrition (EPIC) study. Am J Clin Nutr. 1 abril 2018;107(4):607-16.

4. Castro-Espin C, Agudo A, Bonet C, Katzke V, Turzanski-Fortner R, Aleksandrova K, et al. Inflammatory potential of the diet and risk of breast cancer in the European Investigation into Cancer and Nutrition (EPIC) study. Eur J Epidemiol. setembre 2021;36(9):953-64.

5. Castro-Espin C, Bonet C, Crous-Bou M, Katzke V, Le Cornet C, Jannasch F, et al. Dietary patterns related to biological mechanisms and survival after breast cancer diagnosis: results from a cohort study. Br J Cancer. 3 febrer 2023;
